# Assessment of EMF Human Exposure Levels Due to Wearable Antennas at 5G Frequency Band

**DOI:** 10.3390/s23010104

**Published:** 2022-12-22

**Authors:** Silvia Gallucci, Marta Bonato, Martina Benini, Emma Chiaramello, Serena Fiocchi, Gabriella Tognola, Marta Parazzini

**Affiliations:** 1 National Research Council (CNR), Institute of Electronics, Information Engineering and Telecommunication (IEIIT), 20133 Milano, Italy; 2 Department of Electronics, Information and Bioengineering (DEIB), Politecnico di Milano, 20133 Milano, Italy

**Keywords:** RF-EMF, wearable device, 5G technology, computational dosimetry, realistic human models

## Abstract

(1) Background: This work aims to assess human exposure to EMF due to two different wearable antennas tuned to two 5G bands. (2) Methods: The first one was centered in the lower 5G band, around f = 3.5 GHz, whereas the second one was tuned to the upper 5G band, at 26.5 GHz. Both antennas were positioned on the trunk of four simulated human models. The exposure assessment was performed by electromagnetic numerical simulations. Exposure levels were assessed by quantifying the specific absorption rate averaged on 10 g of tissue (SAR_10g_) and the absorbed power density (S_ab_), depending on the frequency of the wearable antenna. (3) Results: the higher exposure values that resulted were always mainly concentrated in a superficial area just below the antenna itself. In addition, these resulting distributions were narrowed around their peak values and tended to flatten toward lower values in farther anatomical body regions. All the exposure levels complied with ICNIRP guidelines when considering realistic input power. (4) Conclusions: This work highlights the importance of performing an exposure assessment when the antenna is placed on the human wearer, considering the growth of wearable technology and its wide variety of application, particularly regarding future 5G networks.

## 1. Introduction

In recent years, the use of wearable devices has been growing constantly. To date, wearable device communications have been characterized through specific communication protocols, such as Bluetooth and ZigBee [1]. However, in recent years, the fields of application for wearable devices have been increasing, from medical monitoring to military communication [2,3,4,5]. In particular, most interest has moved towards health applications, with the constant monitoring of the health of a patient equipped with a so-called body sensor network (BSN), a network of sensing devices that are placed on the user’s body, able to monitor and collect the vital parameters of the human being, and connect and transmit the data to a mobile coordinator unit [6]. For example, the physiological real-time monitoring of the human user can be performed by the employment of sensors mounted on the user’s body for detecting the respiration rate or the heart rate [7,8,9,10,11]. The wireless body area network (WBAN) is a subgroup of the BSN defined in the IEEE 802.15.6 as a communication standard consisting of low power devices for communication in and around the human body, for medical and non-medical applications [12]. Inside the network, two different communications are possible: between the sensors, and between the central node of the network and an external access point [13].

In addition to the canonical applications of wearable devices mentioned above, wearable networks have also recently included 5G technology, since the use of the 5G protocol permits, for example, the possibility of the involvement of augmented, mixed and virtual realities [14]. For this reason, 5G bands will be involved in wearable communication, including the sub-6 GHz band and the mm-wave band (>24 GHz) [15]. Indeed, some studies are aimed at developing 5G wearable antenna that are multi-band in order to cover the entire 5G spectrum [16].

Since wearable devices are necessarily positioned on the human body, the question of the power absorbed by human tissues is crucial. In this regard, some studies in literature have focused on the assessment of human exposure due to this type of device [17,18]. Furthermore, to reduce human exposure, the wearable antennas are often equipped with an electromagnetic band gap (EBG), which is a type of shielding able to increase the performance of the antenna in terms of improvement in the gain, bandwidth, and backwards radiation suppression [19].

However, to best of our knowledge, the scientific literature is lacking in studies regarding human exposure assessment in scenarios with wearable 5G antennas. The present work is inserted in this context, since this study assesses, for the first time, the human exposure due to two different wearable antennas where each is tuned to a 5G band (sub-6 GHz and >24 GHz band). Therefore, the novelty of this work is the evaluation of exposure due to wearable antennas tuned to 5G bands on simulated realistically anatomical human models, attempting to fill the lack of studies in the literature. Moreover, variations in exposure due to the use of two different frequencies and different human models was deepened in the present work, highlighting the influence of these parameters on human exposure.

From the point of view of the simulated scenarios, both antennas were positioned on the trunk of four simulated human models to assess any differences caused by not only the variability of the human models, but also the variation of the central frequency of the antenna. Since the use of appropriate detailed voxel models is needed for the calculation of the quantities related to human exposure [20], this study was developed using, for the first time, simulated realistic humans as models, with different genders and ages. Moreover, an EBG structure was mounted on the antenna centered at the higher frequency to verify if and how, in this specific analyzed case, the human exposure changed and, more specifically, decreased.

## 2. Materials and Methods

In this section, the two wearable antennas used as EMF sources will be presented. Both antennas were used for off-body communications and were tuned to the 5G spectrum.

### 2.1. Antenna Design

**Figure 1:**
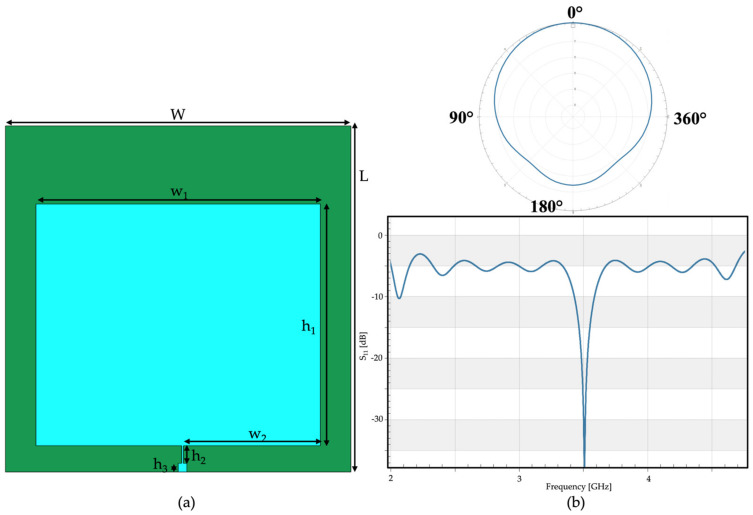
First wearable patch antenna simulated, tuned to f = 3.5 GHz: (**a**) geometry of the antenna, front view; (**b**) performances of the antenna in terms of, in the upper part, radiation pattern (φ = 0°), and, in the lower part, the reflection coefficient (S_11_).

The simulated antennas were tuned to two 5G bands. The first (Figure 1) was centered in the lower 5G band, around f = 3.5 GHz. The geometry of the antenna was based on a single-patch structure with a substrate layer, a ground layer, and a radiating element. The ground, placed on the back of the antenna, was a thin layer made of copper (ε_r_ = 1, σ = 5.813 × 107 S/m) as well as the layer of the radiating element. The patch element was made of polyester (ε_r_ = 2.2, σ = 4.6785 × 10^−8^ S/m). The size of the antenna was defined according to the characteristic frequency of the antenna itself, as summarized in Table 1. The overall size of the antenna was 40 × 40 mm.

The antenna was simulated in free space in order to evaluate its performance in unloaded conditions. In Figure 1b, the radiation pattern and the reflection coefficient (S_11_) show that the performances of the antenna agreed with the typical specifications of a 5G antenna.

**Figure 2:**
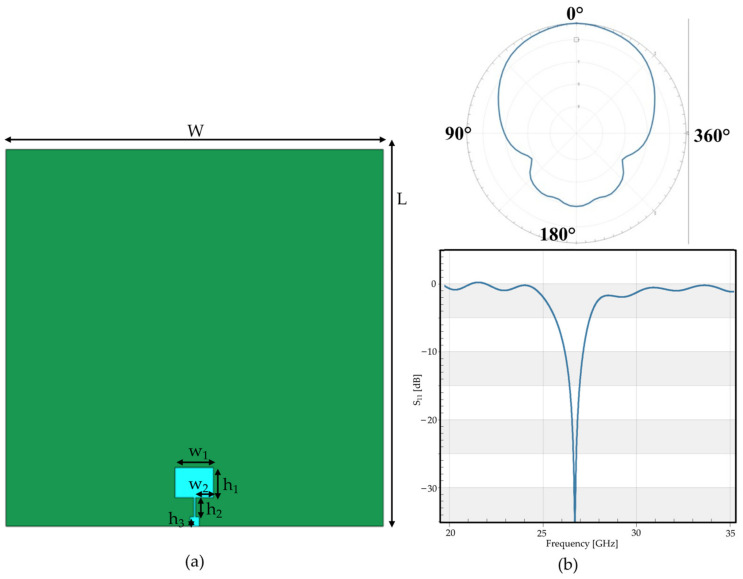
First wearable patch antenna simulated, tuned to f = 26.5 GHz: (**a**) geometry of the antenna: front view; (**b**) performances of the antenna in terms of, in the upper part, radiation pattern (φ = 0°) and, in the lower part, the reflection coefficient (S_11_).

Similar to the first antenna, the second antenna (Figure 2) was a single-patch antenna, composed of a substrate of polyester, and the ground and radiating elements were simulated as a thin layer of copper. The antenna was tuned to the upper 5G band ranging from 24.5 GHz to 27.5 GHz.

The overall size of the second antenna was the same as the first. Table 2 reports the detailed dimensions of the antenna. Moreover, this antenna was also simulated in free space; indeed, the performances of the antenna in terms of reflection coefficient (S_11_) and radiation pattern are reported in Figure 2b, showing good agreement with expectations.

**Figure 3:**
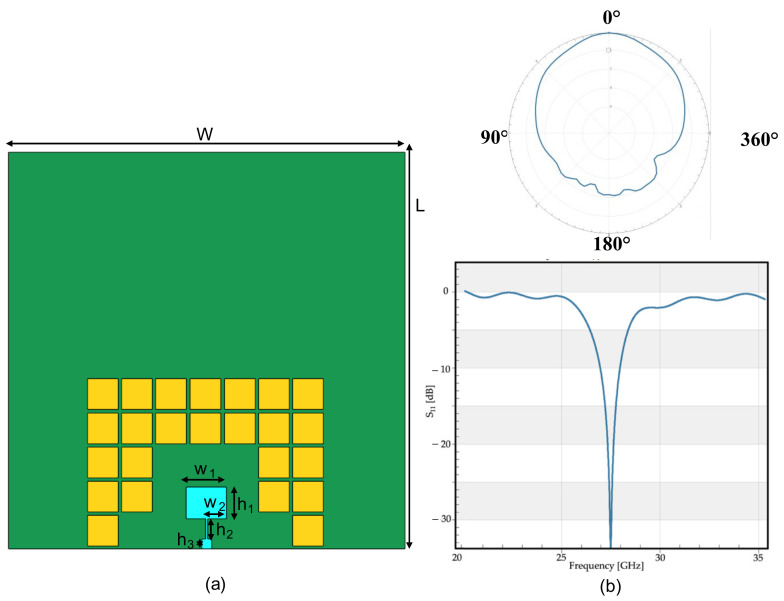
Second wearable patch antenna equipped with the simulated EBG: (**a**) geometry of the antenna, front view; (**b**) performances of the antenna in terms of, in the upper part, radiation pattern (φ = 0°) and, in the lower part, the reflection coefficient (S_11_).

The antenna tuned to the upper band of the 5G spectrum was also equipped with an electromagnetic bandgap (EBG) modeled as a mushroom pattern, as shown in Figure 3. The specific structure of the EBG was based on the study of El May et al. [21], and it was structured as a sequence of thin layers of copper sized 3.1 × 3.1 mm. The radiation pattern and the reflection coefficient (S_11_) of the antenna equipped with the EBG are summarized in Figure 3.

Comparing the radiation pattern of the second antenna with and without the EBG, it was noteworthy that the magnitude of the reflection coefficient was almost the same, the peak was slightly shifted, and the radiation pattern was more directive in the case of the EBG, than the antenna without shielding; in more detail, the value of the gain of the antenna without the EBG was calculated as 7.56 dBi, whereas the gain of the antenna equipped with the bandgap was 8.98 dBi. These values showed that the antenna with the EBG performed better than the one without it. Moreover, it was evident from the radiation patterns that the magnitude of the backscattering decreased when the antenna was equipped with the EBG, again showing good agreement with expectations.

### 2.2. Exposure Simulations

Computational simulations were performed by means of the finite difference time domain (FDTD) method implemented in the simulation platform Sim4Life v.6.2 (ZMT Zurich Med Tech AG, Zurich, Switzerland, www.zurichmedtech.com, accessed on 15 December 2022) to solve the Maxwell’s equations [22].

Simulated human models, namely, a male and female adult model, a male adolescent model, and a female adolescent model, were used from Virtual Population [23]. More specifically, the adult male model was Duke (age = 34 years old, height = 1.77 m, weight = 70.2 kg, BMI = 22.4 kg/m^2^), the adult female model was Ella (age = 26 years old, height = 1.63 m, weight = 57.3 kg, BMI = 21.6 kg/m^2^), the adolescent male model was Louis (age = 14 years old, height = 1.68 m, weight = 49.7 kg, BMI = 17.6 kg/m^2^) and the adolescent female model was Billie (age = 11 years old, height = 1.49 m, weight = 34 kg, BMI = 15.3 kg/m^2^).

**Figure 4:**
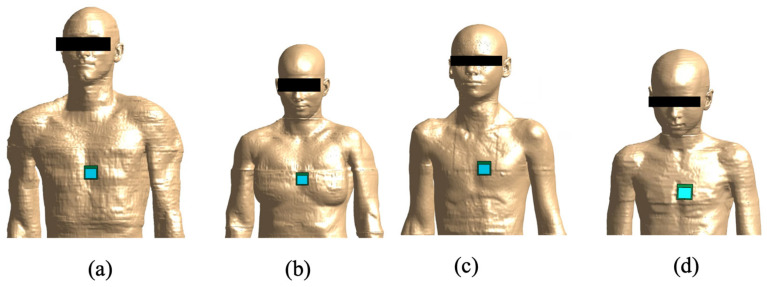
Overview of the simulated exposure configurations when the antenna tuned to 3.5 GHz is positioned on the model: (**a**) Duke, (**b**) Ella, (**c**) Louis, and (**d**) Billie.

For all human models, the antennas were positioned at the same position: on the trunk, centered on the heart of each model (Figure 4). The distance between the antenna and the human model was set equal to 2 mm to simulate a scenario of common use of a wearable device in an off-body configuration that was as realistic as possible. The antenna was positioned with the ground plane facing the human model [24].

Furthermore, the computational domain was discretized with a non-uniform grid with a step ranging from 0.005 mm to 1 mm. The boundary conditions were set as absorbing conditions with the perfect matched layer. The tissue dielectric properties of the human models were chosen according to [25,26,27], considering the chosen frequencies of 3.5 GHz and 26.5 GHz.

### 2.3. Exposure Assessment

To evaluate the interaction between the EMF emitted by the simulated wearable antennas and human tissues, the specific absorption rate (SAR) and the absorbed power density (S_ab_) were evaluated according to ICNIRP guidelines [28]. In more detail, the SAR was calculated as averaged over a cubical mass of 10 g (SAR_10g_), and the S_ab_ was averaged over a square 4 cm^2^ surface area over the skin, that is, the most superficial tissue. Both SAR_10g_ and S_ab_ were obtained with the input power to the antenna set as 1 W. The choice to estimate the SAR over the S_ab_ and vice versa depended on the frequency of the wearable antenna.

All the values of the considered parameter of interest were extracted from a cubic box with dimensions 250 × 250 × 250 mm centered around the antenna; its size and position were defined with the aim of obtaining a cubical box capable of including all the significant values of the parameter of interest in the computational domain, also containing the first slice of the human tissues in which the values were null, following an approach already used in [18].

Moreover, to better quantify the exposure, the descriptive statistics (minimum, 25th, 50th, 75th percentile, and maximum) of the SAR_10g_ and S_ab_ distributions into the box were calculated. The values of both the SAR_10g_ and S_ab_ obtained with the input power to the antenna set as 1 W were compared with the limits imposed by the ICNIRP guidelines [28] for the general public, in the corresponding frequency range.

Finally, for exposure due to the first antenna (f = 3.5 GHz), we calculated the percentage of tissue with SAR_10g_ higher than the threshold (SAR_10gT_). This SAR_10gT_ was defined as the value corresponding to a decrease of 3 dB with respect to the peak SAR_10g_ value in the cubical box. These percentages are useful to understand how much each tissue is involved in exposure with regard to the volume that it occupies in the cubical box.

## 3. Results

This section describes the results of the exposure assessment. The results relating to all human models will be reported, first those results obtained when the antenna was tuned to f = 3.5 GHz (Scenario 1), and then those relating to exposure when the antenna was tuned to f = 26.5 GHz (Scenario 2) with or without the EBG. All results shown were obtained with an input power of 1 W.

### 3.1. Scenario 1

**Figure 5:**
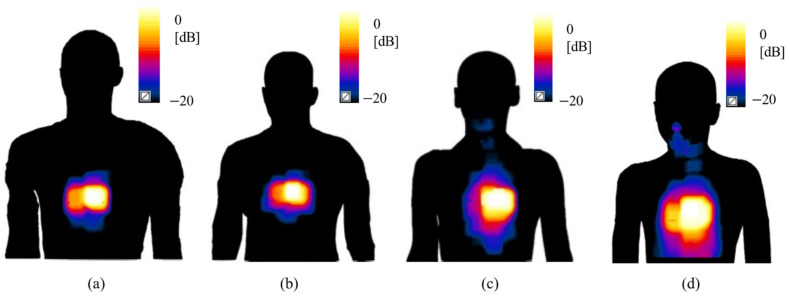
SAR_10g_ distribution on the skin when the antenna is positioned on the trunk of the simulated human models: (**a**) Duke, (**b**) Ella, (**c**) Louis, and (**d**) Billie. All values are represented with respect to the maximum SAR_10g_ value found in each configuration.

Figure 5 shows, as an example, the pattern of SAR_10g_ distributions when the antenna at f = 3.5 GHz was positioned on the trunk of each human model. The distributions were all normalized with respect to their maximum. A qualitative evaluation of these patterns indicated that the region of maximum exposure was mainly concentrated in the portion of the body mainly below the antenna itself, as expected, while the exposure values tended to progressively decrease in the farther body regions.

**Figure 6:**
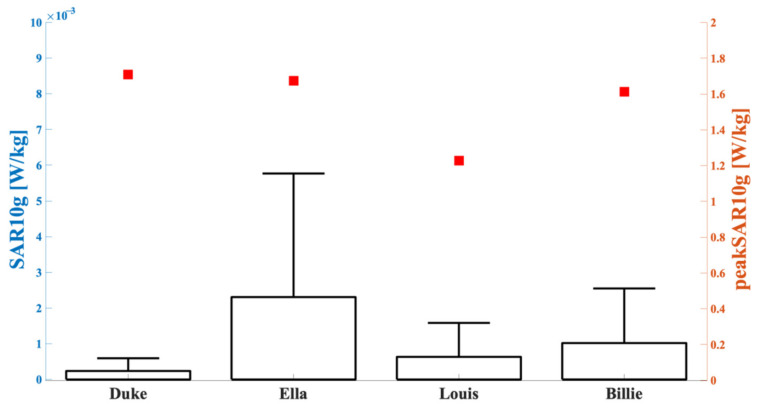
SAR_10g_ (left *y*-axis, boxplot) and peak SAR_10g_ (right *y*-axis, square symbols) in the tissues included in the cubical box relative to the analyzed simulated human model. The bottom and top edges of the boxes are the 25th and 75th percentiles. The lower whisker extends to the minimum values, while the upper whisker extends to 1.5 times the height of the box.

For better characterizing the exposure levels, Figure 6 reports the peak value and boxplot of the SAR_10g_ distributions for all human models. As shown in the figure, slightly higher peak SAR_10g_ values were obtained for the adult models compared with the adolescents. Moreover, the figure clearly shows that for each human model there was a large gap between the peak SAR_10g_ and the 75th percentile of the distribution. It was, therefore, reasonable to assume that the large part of the data of the SAR_10g_ distributions were well below their peak values.

This was, indeed, also confirmed by the percentages of data that were greater than 70% of the peak SAR_10g_, which are summarized in Table 3, together with the peak values of SAR_10g_. The third column shows that less than 1% of SAR_10g_ values were greater than 70% of the peak; among the studied simulated human models, the lowest percentage was found in the Duke model and the highest was found in the Louis model.

**Figure 7:**
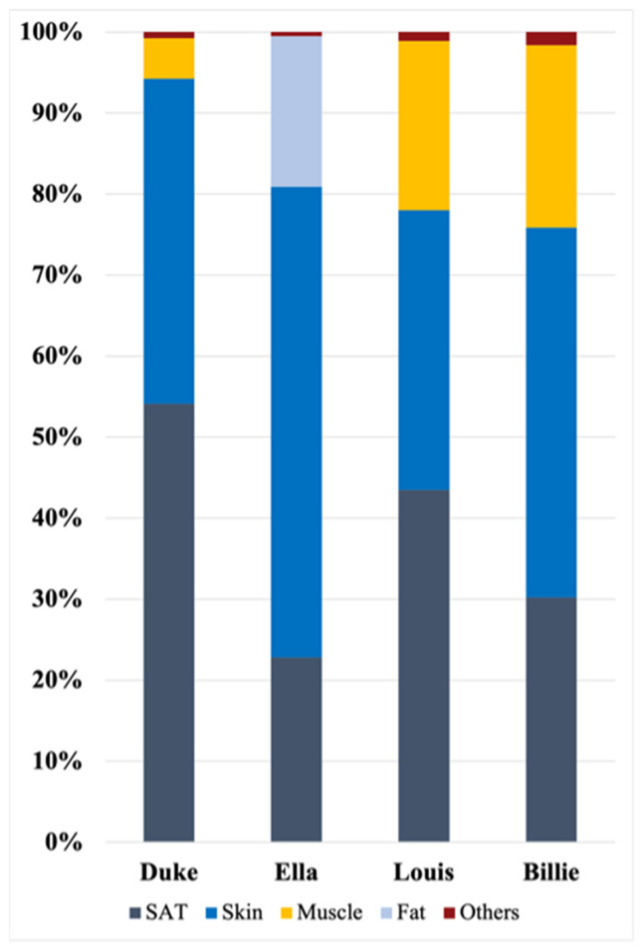
Percentages of tissues most involved in exposure in all studied configurations; the percentages are calculated with respect to the volume that the tissue occupies in the cubical sensor.

Furthermore, Figure 7 represents the percentages of tissues with SAR_10g_ values greater than the threshold, SAR_10gT_. These percentages were obtained by considering the volume that the single tissue occupied within the cubical box centered around the antenna, where the distributions were evaluated. The figure shows that the two tissues mainly involved in exposure across all models were the skin and the subcutaneous tissues (SAT). Taken together, these tissues were responsible for more than 75% of the higher values of exposure level.

### 3.2. Scenario 2

As in the case of Scenario 1, when the antenna at f = 26.5 GHz was positioned on the trunk of each model, the region of higher exposure was mainly concentrated in the portion of the body under the antenna itself, with a progressive decrease in exposure level in the surrounding body regions.

**Figure 8:**
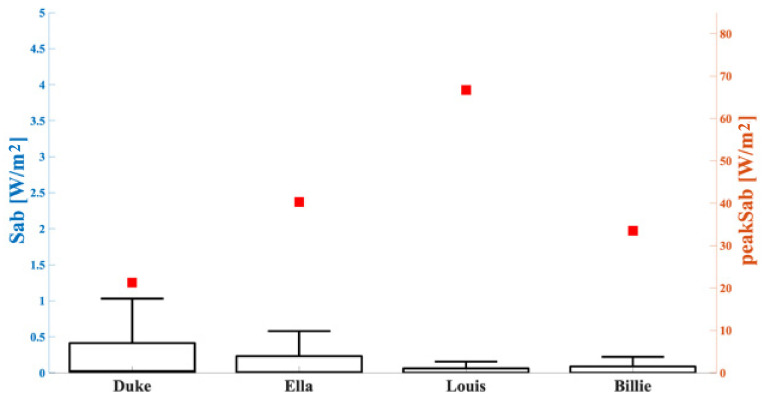
S_ab_ (left *y*-axis, boxplot) and peak S_ab_ (right *y*-axis, square symbols) in the tissues included in the cubical box relative to the analyzed simulated human model. The bottom and top edges of the boxes are the 25th and 75th percentiles. The lower whisker extends to the minimum values, while the upper whisker extends to 1.5 times the height of the box.

The peak values and the descriptive statistics of the S_ab_ distributions over the skin are presented for each human model in Figure 8. The figure shows that higher peak S_ab_ values were obtained for the male adolescent Louis model, whereas the minimum value was observed in the male adult model, Duke.

Additionally, similar to Scenario 1, the huge gap between the maximum value and the 75th percentile was evident, and also confirmed by the percentages of data greater than 70% of the peak value that did not exceed 1% in any analyzed configuration. These percentages and the values of peak S_ab_ for each human model are shown in Table 4.

**Figure 9:**
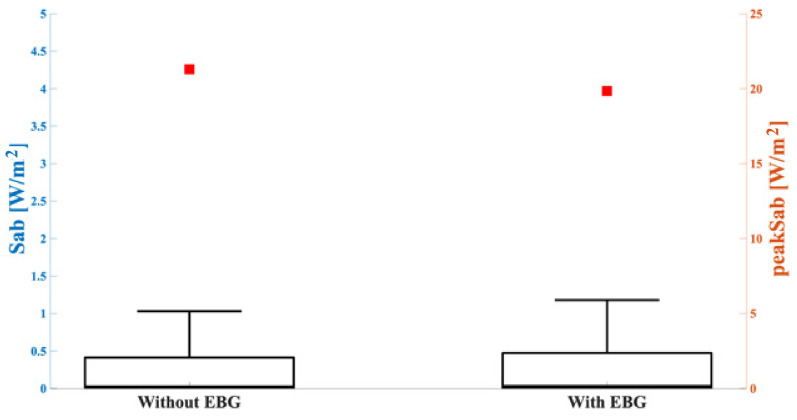
S_ab_ (left *y*-axis, boxplot) and peak S_ab_ (right *y*-axis, square symbols) in the tissues included in the cubical box relative to the configurations without (on the left) and with (on the right) the EBG mounted on the antenna. The bottom and top edges of the boxes are the 25th and 75th percentiles. The lower whisker extends to the minimum values, while the upper whisker extends to 1.5 times the height of the box.

To test if the presence of the EBG was incisive in the human exposure context, we also simulated the antenna at the highest frequency with the EBG and positioned it on the trunk of the Duke model. Then we extracted the S_ab_ values averaged over the skin surface and compared them with the results obtained in the case without the EBG. Figure 9 shows the peak values and the descriptive statistics of the S_ab_ distributions relative to the configurations without, and with, the EBG. Comparing the results obtained in these two cases, it was noteworthy that the most evident difference resided in the peak value of the S_ab_, because the peak value found in the case with the EBG was about 7% lower than without the EBG, whereas the box plots in the two cases were almost identical.

## 4. Discussion

This work aimed to assess the exposure due to two different wearable antennas working in the 5G band, when they are positioned on the trunk of different simulated realistic human models.

First of all, considering the antenna tuned to 3.5 GHz (i.e., Scenario 1) for all human models, the highest exposure values were mainly concentrated in a specific area below the surface of the antenna itself, with the SAR_10g_ distributions narrowed around their peak value. Moreover, the values of the peak SAR_10g_ observed in all the studied configurations were lower than the limits imposed by the ICNIRP guidelines [28]; indeed, considering the limit in terms of local head and trunk SAR_10g_ for the frequency range from 100 kHz to 6 GHz for the general public, i.e., 2 W/kg, our obtained results were below this limit. In fact, the highest value was detected in the Duke model (1.72 W/kg) and the lowest value in the Louis model (1.23 W/kg), which were both below the limit. It is noteworthy that these values, which are compliant with ICNIRP, were obtained with an input power of 1 W, which is higher than a realistic situation, which is in the order of tens of mW [18]. Therefore, in terms of realistic input power, all the exposure levels are well below the limits.

Furthermore, we also studied the distributions of SAR_10g_ values among tissues, namely, the identification of the most involved tissues, by quantifying the percentage of tissue where the SAR_10g_ distribution was higher than the threshold. These percentages clearly showed that, in all the studied configurations, the skin and the subcutaneous adipose tissue (SAT) were the most involved tissues in exposure; the greatest involvement of these tissues was due to the fact that they are the most superficial ones (see Figure 7). It is important to underline that these percentages were given in proportion to the volume that each tissue occupied in the cubical box. In addition, muscle was among the tissues mostly involved in exposure for all human models except for Ella; in that case, fat tissue was characterized by higher exposure than the other simulated human model cases. This was probably due to the larger volume that fat occupies in the volume of interest for the adult female model, compared with other human models.

The results obtained in this work are in line with previous studies in which similar cases were analyzed. In particular, Du et al. [29] studied a multiband antenna positioned on a human phantom, modeled as three layers: skin (2 mm), fat (5 mm) and muscle (20 mm). The antenna was positioned at a distance of 5 mm from the phantom and an input power of 0.2 W was imposed. For the band centered at f = 3.5 GHz, the peak SAR_10g_ was equal to 0.89 W/kg. This result is comparable with the SAR_10g_ obtained in the present study, although the human model was not realistic but stratified, the distance between the antenna and the model was higher, and the input power was lower than 1 W. On the other hand, the antenna was compact coplanar waveguide (CPW)-fed, therefore, there was no shielding between the antenna and the exposed model. Taking all these aspects together, it is considered reasonable that the results are in line. Moreover, El May et al. [30] showed that a 5G wearable antenna, tuned to f = 3.5 GHz, fed with an input power of 0.5 W, and positioned at a distance of 1 mm from a multilayer rectangular human tissue healthy model (skin, fat, and muscle) resulted in a peak SAR_10g_ equal to 8.75 W/kg. This higher SAR_10g_ value was probably because the ground plane of the antenna was not facing the user, as in our exposure scenario. Finally, Anbarasu et al. [31] and Liao et al. [32] proposed two studies with multiple-input multiple-output (MIMO) wearable antenna tuned to the sub-6 GHz 5G band. In the first study [31], the MIMO antenna for a smartwatch application was positioned on a phantom mimicking a hand, made of skin, obtaining a peak SAR_10g_ varying from 0.272 W/kg up to 0.306 W/kg, depending on the configuration of use of the antenna. The second study [32] showed an antenna for a smartwatch simulated on a human wrist phantom made as a parallelepiped of four tissue layers: from outside to the inside, skin (0.65 mm), fat (4.55 mm), muscle (1.3 mm), and bone (26 mm). The antenna was placed 2 mm away from the phantom. The peak SAR_10g_ values obtained by varying the configuration of use of the antenna ranged from 0.67 W/kg up to 0.98 W/kg.

Considering the antenna tuned to 26.5 GHz (i.e., Scenario 2), the extracted parameter of interest was the absorbed power density (S_ab_) over the skin, according to the ICNIRP guidelines [28]; it is important to highlight that only the most superficial tissue was studied in this case because of the low penetration depth due to the high frequency considered. In this scenario, the highest value of peak S_ab_ was found in the Louis model (66.7 W/m^2^), whereas the lowest was found in the Duke model (21.3 W/m^2^). A possible explanation for that was the lower thickness of the skin in the Louis model, compared with the others. As for Scenario 1, in Scenario 2 the regions with the highest exposure values were mainly concentrated in a specific area below the antenna itself, with S_ab_ distributions narrowed around their peak value for all human models. With the reference power input 1 W used in the simulations, the absorbed power density peaks in the skin in all the configurations were always over the value of 20 W/m^2^, indicated as the limit in the ICNIRP guidelines for the general public. However, as before, it is important to underline that the maximum input power of the wearable antennas in real applications is around tens of mW [18]. The use of these input powers would greatly reduce the simulated values of exposure, thereby complying with the indicated ICNIRP guideline limits.

Finally, in order to know if the presence of an EBG could modify the exposure due to the wearable device, we simulated the same antenna with and without the EBG. As Figure 9 shows, there was no noticeable difference between the presence, or not, of the EBG in this simulated scenario, even if the radiation patterns were slightly different between each other (see Figure 2 and Figure 3); in fact, the peak S_ab_ in the case without the EBG was only slightly higher than with the EBG (about 7% difference). It must be highlighted that, in this specific case, the ground plane facing the user in both cases strongly reduced the impact of the EMF in terms of human exposure [33], so, the shielding effect was reduced due to the EBG only.

In contrast with Scenario 1, there still seems to be a lack of work in the literature regarding the assessment of exposure due to a 5G wearable device, although the body area network could be a good environment in which 5G technology could be applied [16]. Due to this, it is not possible to make a direct comparison of our results with previous studies that assessed human exposure to 5G wearable antennas. It is, however, interesting to present at least two studies that designed and preliminarily assessed human exposure due to two wearable antennas at 5G frequencies. In the first study [34], the antenna was a finger ring phased antenna resonant at 28 GHz. The antenna was simulated as inserted on three fingers of the hand of a human model, and the assessment was performed in terms of power loss density; these values ranged from 75.3 dB (W/m^3^) to 75.45 dB (W/m^3^). In the second study [35], the antenna (tuned to f = 28 GHz) was integrated into a smartwatch positioned on the wrist of a human model. In this case, the SAR_10g_ and the SAR_1g_ were estimated, and they were equal to 0.113 W/kg and 0.301 W/kg, respectively, but no results regarding the absorbed power density were shown.

## 5. Conclusions

In conclusion, this work has assessed, for the first time, the exposure of a human to the EMF emitted by wearable antennas, each one tuned to a 5G band (sub-6 GHz and >24 GHz band), positioned on the trunk of four simulated realistic human models. For all the configurations analyzed, the higher exposure values that resulted were mainly concentrated on a superficial area immediately below the antenna itself. Moreover, these distributions were narrowed around their peak values and tended to flatten toward lower values in the farther anatomical regions. Finally, all the exposure levels complied with ICNIRP guidelines, when evaluated considering realistic input power.

## Figures and Tables

**Table 1:** Size of the antenna tuned to f = 3.5 GHz.

Values	Dimensions (mm)
W	40
L	40
w_1_	32.92
w_2_	15.86
h_1_	27.93
h_2_	2.04
h_3_	1

**Table 2:** Size of the antenna tuned to f = 26.5 GHz.

Values	Dimensions (mm)
W	40
L	40
w_1_	4.54
w_2_	2.19
h_1_	3.664
h_2_	2.04
h_3_	1

**Table 3:** Peak SAR_10g_ and percentage of SAR_10g_ values greater than the 70% of the peak SAR_10g_ for each configuration.

Human Model	Peak SAR_10g_ (W/kg)	Values Greater than 70% of Peak SAR_10g_ (W/kg)
Duke	1.72	0.45
Ella	1.67	0.55
Louis	1.23	0.78
Billie	1.61	0.72

**Table 4:** Peak S_ab_ and percentage of S_ab_ values greater than 70% of peak S_ab_ for each configuration.

Human Model	Peak S_ab_ (W/m^2^)	Values Greater than 70% of Peak S_ab_ (W/m^2^)
Duke	21.3	0.45
Ella	40.3	0.30
Louis	66.7	0.27
Billie	33.5	0.18

## References

[1] Meharouech A., Jocelyn E., Mehaoua A. (2019). Moving Towards Body-to-Body Sensor Networks for Ubiquitous Applications: A Survey. J. Sens. Actuator Netw..

[2] Lee H., Tak J., Choi J. (2017). Wearable Antenna Integrated into Military Berets for Indoor/Outdoor Positioning System. IEEE Antennas Wirel. Propag. Lett..

[3] Paracha K.N., Rahim S.K.A., Soh P.J., Khalily M. (2019). Wearable Antennas: A Review of Material, Structures and Innovative Features for Autonomous Communication and Sensing. IEEE Access.

[4] Chahat N., Zhadobov M., Le Coq L., Sauleau R. (2012). Wearable Endfire Textile Antenna for On-Body Communications at 60 GHz. IEEE Antennas Wirel. Propag. Lett..

[5] Chahat N., Zhadobov M., Sauleau R., Mahdjoubi K. Improvement of the On-Body Performance of a Dual-Band Textile Antenna Using an EBG Structure. Proceedings of the 2010 Loughborough Antennas & Propagation Conference.

[6] Gravina R., Fortino G. (2020). Wearable Body Sensor Networks: State-of-the-Art and Research Directions. IEEE Sens. J..

[7] Ahadi M., Roudjane M., Dugas M.A., Miled A., Messadeq Y. (2022). Wearable Sensor Based on Flexible Sinusoidal Antenna for Strain Sensing Applications. Sensors.

[8] De Fazio R., Stabile M., De Vittorio R., Velazquez R., Visconti P. (2021). An Overview of Wearable Piezoresistive and Inertial Sensors for Respiration Rate Monitoring. Electronics.

[9] Wagih M., Malik O., Weddell A.S., Beeby S. (2022). E-Textile Breathing Sensor Using Fully Textile Wearable Antennas. Eng. Proc..

[10] El Garbi M., Fernandez-Garcia R., Gil I. (2022). Embroidered Wearable Antenna-Based Sensor for Real-Time Breath Monitoring. Measurements.

[11] Bent B., Goldstein B.A., Kibbe W.A., Dunn J.P. (2020). Investigating Sources of Inaccuracy in Wearable Optical Heart Rate Sensors. Digit. Med..

[12] (2012). IEEE Standard for Local and Metropolitan Area Networks part 15.6: Wireless Body Area Networks.

[13] Punj R., Kumar R. (2019). Technology Aspects of WBANs for Health Monitoring: A Comprehensive Review. Wirel. Netw..

[14] Ometov A., Shubina V., Klus L., Skibinska J., Saafi J., Pascacio P., Lohan E.S. (2021). A Survey on Wearable Technology: History, State-of-the-Art and Current Challenges. Comput. Netw..

[15] Pirmagomedov R., Koucheryavy Y. (2021). IoT Technologies for Augmented Human: A Survey. Internet Things.

[16] Kumar A., Mahto S.K., Sinha R., Choubey A. (2021). Dual Circular Slot Ring Triple-Band MIMO Antenna for 5G Applications. Frequenz.

[17] Gallucci S., Bonato M., Chiaramello E., Fiocchi S., Tognola G., Parazzini M. (2022). Human Exposure Assessment to Wearable Antennas: Effect of Position and Interindividual Anatomical Variability. Int. J. Environ. Res. Public Health.

[18] Wissem E.M., Sfar I., Osman L., Ribero J.M. (2021). A Textile EBG-Based Antenna for Future 5G-IoT Millimeter-Wave Applications. Electronics.

[19] Ali U., Ullah J., Khan M., Shafi B., Kamal A., Basir A., Seagar R.D. (2017). Design and SAR Analysis of Wearable Antenna on Various Parts of Human Body, Using Conventional and Artificial Ground Planes. J. Electr. Eng. Technol..

[20] Cousin R., Rutschlin M., Wittig T., Bhattacharya A. (2015). Simulation of Wearable Antennas for Body Centric Wireless Communication. Sens. Imaging.

[21] El May W., Sfar J., Ribero J.M., Osman L. A Millimeter-Wave Textile Antenna Loaded with EBG Structures for 5G and IoT Applications. Proceedings of the 2019 IEEE 19th Mediterranean Microwave Symposium (MMS).

[22] Taflove A., Hagness S.C., Piket-May M. (2005). Computational Electromagnetics: The Finite-Difference Time-Domain Method. Electr. Eng. Handb..

[23] Gosselin M.C., Neufeld E., Moser H., Huber E., Farcito S., Gerber L., Kuster N. (2014). Development of a New Generation of High-Resolution Anatomical Models for Medical Device Evaluation: The Virtual Population 3.0. Phys. Med. Biol..

[24] Bait-Suwailam M.M., Labiano I., Alomainy A. (2020). Impedance Enhancement of Textile Grounded Loop Antenna Using High-Impedance Surface (HIS) for Healthcare Applications. Sensors.

[25] Gabriel C., Gabriel S., Corthout Y.E. (1996). The Dielectric Properties of Biological Tissues: I. Literature Survey. Phys. Med. Biol..

[26] Gabriel S., Lau R.W., Gabriel C. (1996). The Dielectric Properties of Biological Tissues: II. Measurements in the Frequency Range 10 Hz to 20 GHz. Phys. Med. Biol..

[27] Gabriel S., Lau R.W., Gabriel C. (1996). The dielectric properties of biological tissues: III. Parametric models for the dielectric spectrum of tissues. Phys. Med. Biol..

[28] International Commission on Non-Ionizing Radiation and Protection (2020). ICNIRP Guidelines for Limiting Exposure to Time-Varying Electric, Magnetic and Electromagnetic Fields (up to 300 GHz). Health Phys..

[29] Du C., Li X., Zhong S. (2019). Compact Liquid Crystal Polymer Based Tri-Band Flexible Antenna for ELAN/WiMAX/5G Applications. IEEE Access.

[30] El May W., Sfar I., Robeiro J.M., Osman L. (2021). Design of Low-Profile and Safe Low SAR Tri-Band Textile EBG-Based Antenna for IoT Applications. Prog. Electromagn. Res. Lett..

[31] Anbarasu M., Nithiyanantham J. (2021). Performance Analysis of Highly Efficient Two-Port MIMO Antenna for 5G Wearable Applications. IETE J. Res..

[32] Liao C.T., Yang Z.K., Chen H.M. (2021). Multiple Integrated Antennas for Wearable Fifth-Generation Communication and Internet of Things Applications. IEEE Access.

[33] Seimeni M.S., Tsolis A., Alexandridis A.A., Pantelopoulos S.A. (2021). Human Exposure to EMFs from Wearable Textile Patch Antennas: Experimental Evaluation of the Ground-Plane Effect. Prog. Electromagn. Res. B.

[34] Syrytsin I., Zhang S., Pedersen G.F. Finger Ring Phased Antenna for 5G IoT and Sensor Networks at 28 GHz. Proceedings of the 12th European Conference on Antennas and Propagation (EuCAP 2018).

[35] Ahmed M.I., Ahmed M.F. Design of 5G Smart Watch with Millimeter Wave Wearable Antenna. Proceedings of the 2019 7th International Japan-Africa Conference on Electronics, Communications, and Computations (JAC-ECC).

